# Supplementary material for: Gradient-mixing LEGO robots for purifying DNA origami nanostructures of multiple components by rate-zonal centrifugation
Source: PLoS One. 2023 Jul 19;18(7):e0283134. doi: 10.1371/journal.pone.0283134 (PMC10355445; doi:10.1371/journal.pone.0283134)
Supplement: S1 File — (PDF) [file pone.0283134.s001.pdf]

Supporting Information

Gradient-mixing LEGO robots for purifying DNA origami nanostructures of multiple components by rate-zonal centrifugation

Jason Sentosa[a,b,1], **Franky Djutanta**[a,c,1,2], **Brian Horne**[d,1], **Dominic Showkeir**[d,1], **Robert Rezvani**[a,c], **Chloe Leff**[a,e], **Swechchha Pradhan**[a,c], **and Rizal F. Hariadi** [a,e,2]

[a]Biodesign Center for Molecular Design and Biomimetics (at the Biodesign Institute) at Arizona State University, Tempe, AZ 85287, USA.; [b]Department of Biomedical Engineering, Georgia Institute of Technology, GA; [c]School for Engineering of Matter, Transport and Energy, Arizona State University, AZ.; [d]Department of Physics, Arizona State University, AZ.; [e]School of Molecular Sciences, Arizona State University, AZ.

[1]These authors contributed equally.
[2]To whom correspondence should be addressed. E-mail: fdjutant@asu.edu AND rhariadi@asu.edu

Contents

S1 Supporting Figures 2
Figure S1: LEGO Building Instructions 2
Figure S2: SYBR Gold Control 3
Figure S3: Gradient Reproducibility 4
Figure S4: Monomer Purification 5
Figure S5: Separation of dimer by RZC using 30−60% (w/v) sucrose gradient 6

S2 Supporting Table 7
Table S1: DNA Sequences 7
Table S2: Cost Analysis 9

S3 Protocols 9
Materials 9
Equipments 9
Protocols 10

S4 Supporting Information Repository 13
Movie S1 13

S1. Supporting Figures

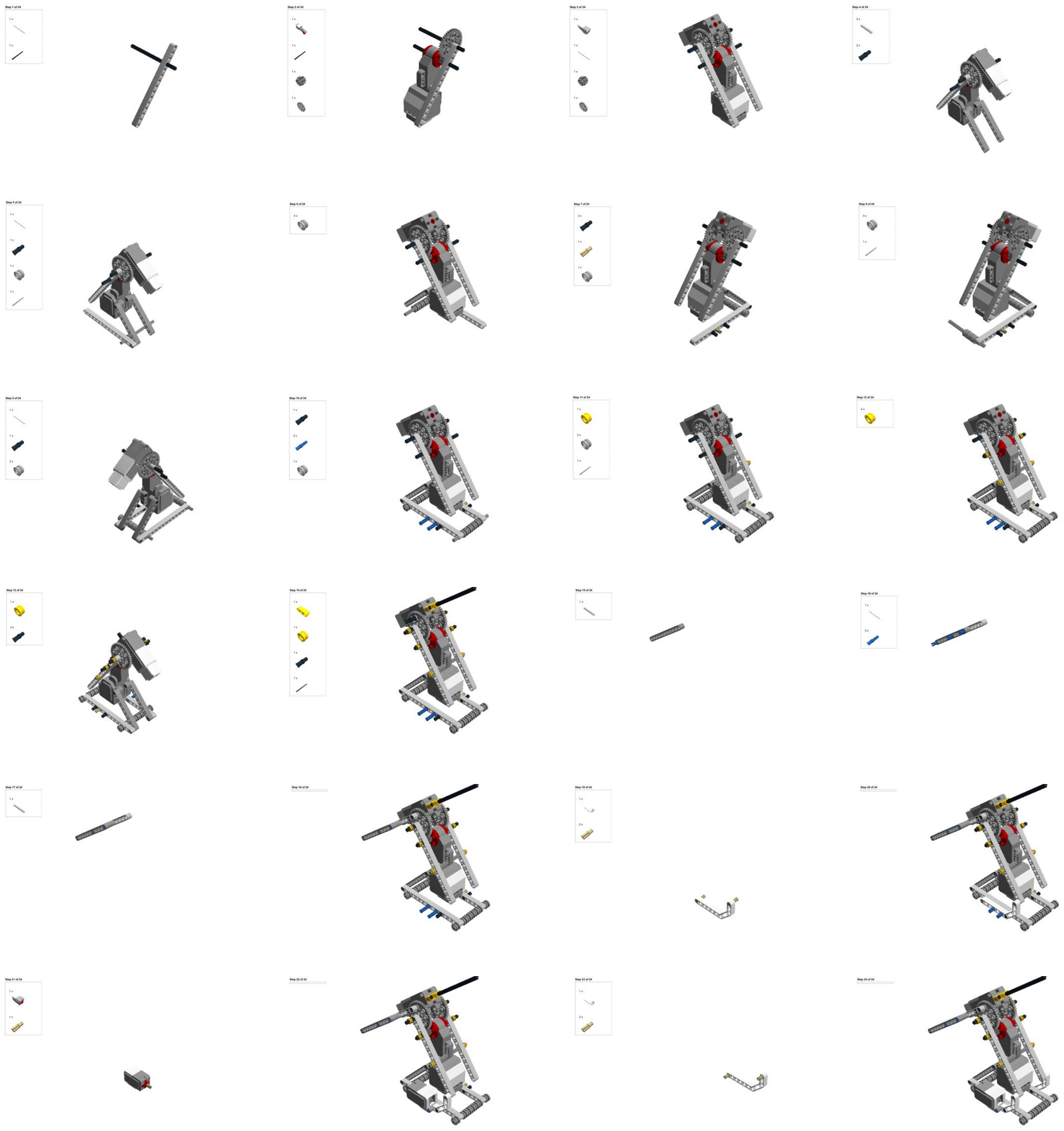

Fig. S1 **Building instructions for the LEGO gradient mixer**. There are 24 steps in total, excluding the 3D printed centrifuge tube holder. An animation showing the build instructions can be found in Supporting Information Repository

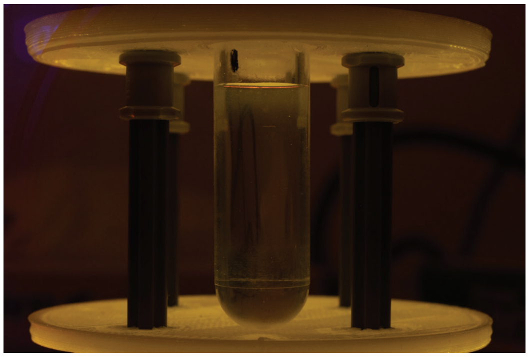

Fig. S2 **RZC of SYBR gold-only sample using 30–60% (v/v) glycerol gradient**. The sample was centrifuged at 50,000 rpm at 4°C for 3 hours. The SYBR gold was undetectable under blue LED illumination.

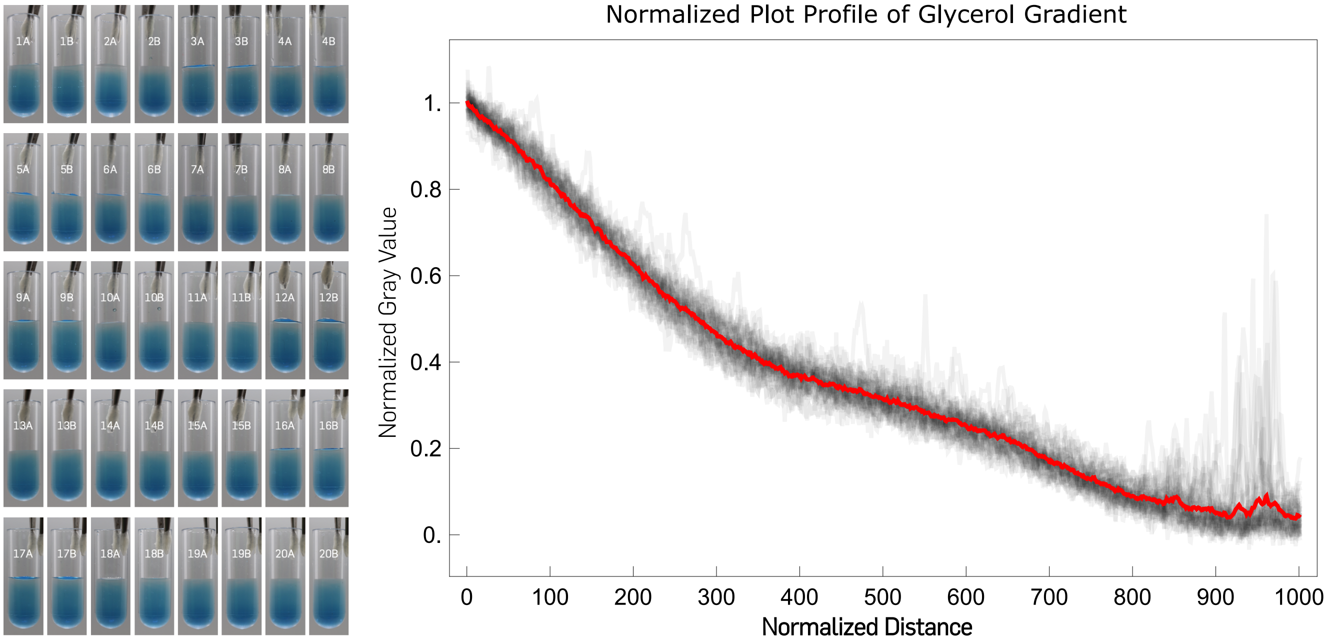

Fig. S3 **Reproducibility analysis for LEGO gradient mixer.** (Left) The LEGO gradient mixer was tested for consistency by repeating a 60 sec 15% to 45% gradient mixing 20 times. Each resulting gradient of glycerol was photographed twice. The 45 % glycerol was dyed blue as a visual indicator. (Right) Images of the gradients were analyzed in ImageJ. First, we measured the gray gradient value as a function of distance. We then normalized the gray value to a scale of 1 to 0, with 1 being the maximum gray value in a particular plot and 0 is the minimum. To account for different pixel sizes due to the difference in the distance between the camera and the object, we normalize the liquid height inside each ultracentrifuge to 1000 points. After normalizing for the gray value and the distance, a mean plot (red line) was generated by calculating the mean at each x position (red). The mean difference between a given point from the mean (red) was calculated at 4.9±4.7% (N=40).

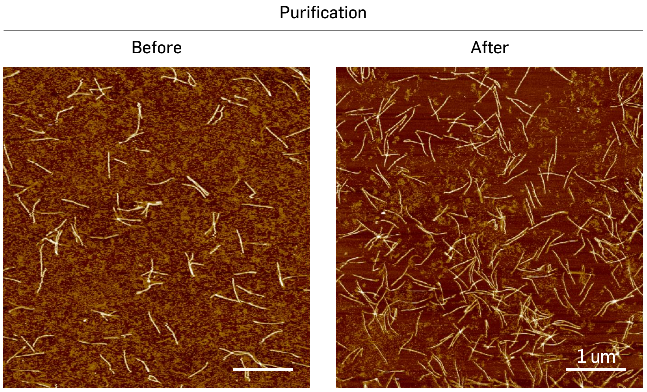

Fig. S4 **Uncropped AFM images of unpurified (left) and purified (right) monomers** corresponding to Figs 4C and D, respectively. Custom code for CDF analysis in `Mathematica` is available in Supporting Information Repository. Scale bar = 1 µm.

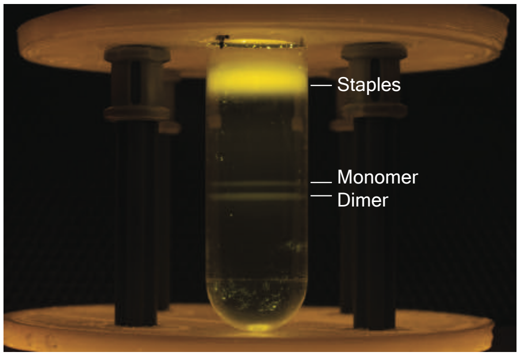

Fig. S5 **Separation of dimers from a mixture of monomers and dimers using 30−60% (w/v) sucrose gradient via RZC using a sucrose concentration gradient of 30−60% (w/v).** The sucrose gradient was created using the LEGO gradient mixer. The sample was centrifuged at 50,000 rpm at 4°C for 6 hours.

S2. Supporting Table

Table S1 Computer-aided staple strand sequences for the DNA origami nanotube monomers.

No	Name	Sequence
1	1[28]-5[41]-edge-ON	GGT CAG GAT TAG AGG AGC AAG AAA CAA TTT TAA GAA AAG TAA
2	2[41]-4[28]-edge-ON	AAA GCG AAC CAG ACT TCA ACA GTT TCA GGT AAA TGA ATT TTC
3	2[1343]-4[1330]-edge-ON	TGT GAG AGA TAG ACT ACG TGA ACC ATC ATA AAG CAC TAA ATC
4	1[1330]-5[1343]-edge-ON	ACC GGA AAC AAT CGT CAA TTA CCT GAG CAC CAA GTT ACA AAA
5	1[28]-5[41]-edge-OFF	TTT TTA CTC CAA CAG GTC AGG ATT AGA GGA GCA AGA AAC AAT TTT AAG AAA AGT AA
6	3[16]-2[19]-edge-OFF	TGC TAA ACA ACT CGG AAG CAA TTT TT
7	2[41]-4[28]-edge-OFF	AAA GCG AAC CAG ACT TCA ACA GTT TCA GGT AAA TGA ATT TTC TGT ATG GGA TTT TTT TT
8	5[12]-0[12]-edge-OFF	TTT TTT CTT ACC GAA GCC CTT GAA ATA GCA ATA GCT ATT TTT
9	2[1343]-4[1330]-edge-OFF	TTT TGT TTA CCA GTC CCG GAA TTT GTG AGA GAT AGA CTA CGT GAA CCA TCA TAA AGC ACT AAA TC
10	0[1355]-1[1362]-edge-OFF	TTT TTG AAT TAT TCA TTG CGA AAC GTA CAG CGC CAT TTT TT
11	1[1330]-5[1343]-edge-OFF	ACC GGA AAC AAT CGT CAA TTA CCT GAG CAC CAA GTT ACA AAA TCG CGC AGA GGC TTT TT
12	4[1360]-3[1360]-edge-OFF	TTT TTG GGG TCG AGG TGC CGC CCA AAT CAA GTT TTT TTT T
13	short 1	TAG AAT TAT TCA TTG CGA AAC GTA CAG CCC AGT CCC GGA ATT
14	short 2	TCG CGC AGA GGC TCG GGT CGA GGT GCC GCC CAA ATC AAG TTT
15	short 3	TTT GCT AAA CAA CTC GGA AGC AAG TTT AGC CAT ACT CCA ACA
16	short 4	TGT ATG GGA TTT TGT TAC CGA AGC CCT TGA AAT AGC AAT AGC
17	0[349]-2[336]	TTA GTT GCT ATT TTA AAT CAT ACA GGC ACT ATC ATA ACC CTC
18	0[685]-2[672]	CGA CAA TAA ACA ACC TGG AGC AAA CAA GAA GAA CCG GAT ATT
19	0[1021]-2[1008]	ATC GCA AGA CAA AGT GGG CGC ATC GTA AGC AAC CGC AAG AAT
20	0[391]-2[378]	AAC CTC CCG ACT TGA AAG CCT CAG AGC AAC ATA ACG CCA AAA
21	0[727]-2[714]	TAT AAA GTA CCG ACA GCA TGT CAA TCA TAG CGC AGT GTC ACT
22	0[1063]-2[1050]	GGT TGG GTT ATA TAC GAC AGT ATC GGC CCC GGA CTT GTA GAA
23	0[433]-2[420]	GAA GGC TTA TCC GGT TAT GAC CCT GTA AAT CAG TTG AGA TTT
24	0[769]-2[756]	TGT AAT TTA GGC AGC CCA AAA ACA GGA ACC TGC AGC CAG CGG
25	0[1105]-2[1092]	AAA TCA TAG GTC TGC CGG CAC CGC TTC TCT GGC AGC CTC CGG
26	0[475]-2[462]	TAG GAA TCA TTA CCA CGC AAG GAT AAA AAA ACG AAC TAA CGG
27	0[811]-2[798]	GTA GGG CTT AAT TGA ACG TTA ATA TTT TGG TGT GTT CAG CAA
28	0[1147]-2[1134]	CTT AGA TTA AGA CGC GCC ATT CAG GCT GAT TGC CGT TCC GGC
29	0[517]-2[504]	CCG CAC TCA TCG AGG CAA TGC CTG AGT ATT ATA CCA GTC AGG
30	0[853]-2[840]	GCG TTA TAC AAA TTA TCA GCT CAT TTT TTG GGT AAA GGT TTC
31	0[1189]-2[1176]	TTA ATT AAT TTT CCG GGC CTC TTC GCT ACA GGC GGC CTT TAG
32	0[559]-2[546]	TGT CTT TCC TTA TCG AAA GGC CGG AGA CAA TCA TTG TGA ATT
33	0[895]-2[882]	CCG GAA TCA TAA TTT TCG CGT CTG GCC TGT TGC GGT ATG AGC
34	0[1231]-2[1218]	ATA TAT GTG AGT GAC TGC AAG GCG ATT ATT GTG TAC ATC GAC
35	0[601]-2[588]	CAT CCT AAT TTA CGA CCG TTC TAG CTG AAC GAG TAG TAA ATT
36	0[937]-2[924]	TAC CGA CCG TGT GAA TGT GAG CGA GTA AGG GGT CAT TGC AGG
37	0[1273]-2[1260]	TCA TTT GAA TTA CCC ACG ACG TTG TAA ACA AAC TTA AAT TTC
38	0[643]-2[630]	TTA TCA ACA ATA GAT TTG AGA GAT CTA CCT CAT TCA GTG AAT
39	0[979]-2[966]	TTA GTT AAT TTC ATA CGG CGG ATT GAC CTC CCA CGC AAC CAG
40	0[1315]-2[1302]	AAA CAA ACA TCA AGT GGA GCC GCC ACG GAG GGA TAG CTC TCA
41	3[896]-1[909]	CAT AAA GTG TAA AGA TCC GCC GGG CGC GTC CTG TAG CCA GCT
42	3[1232]-1[1245]	GTT GAG TGT TGT TCC AGC AGT TGG GCG GAG TTG GGT AAC GCC
43	4[279]-0[266]	AAT AGG TGT ATC ACA GAC AAA AGG GCG AAG CCT AAT TTG CCA
44	4[615]-0[602]	GTC AGA CGA TTG GCC TCC CTC AGA GCC GGA AAA ATA ATA TCC
45	4[951]-0[938]	GTA ATA ACA TCA CTG CCG TCA ATA GAT ATT AAT GGT TTG AAA
46	4[1287]-0[1274]	AGC CGG CGA ACG TGA ACA GTA CCT TTT ATA CAT TTA ACA ATT
47	5[294]-3[307]	AGG GAG GGA AGG TAC GAG AGG GTT GAT ATC ACC CTC AGC AGC
48	5[630]-3[643]	GCC ACC CTC AGA GCC GCC AGC ATT GAC AAA GAG GAC AGA TGA
49	5[966]-3[979]	TTA GAA GTA TTA GAA ACC GTT GTA GCA AGC TTT CCA GTC GGG
50	5[1302]-3[1315]	ATA ACG GAT TCG CCA GCC CCC GAT TTA GAA AAC CGT CTA TCA
51	3[938]-1[951]	CAC ATT AAT TGC GTT GTC CAG CAT CAG CCA ACC CGT CGG ATT
52	3[1274]-1[1287]	GAA CGT GGA CTC CAC AGA AAC AGC GGA TAC GAC GGC CAG TGC
53	4[321]-0[308]	CGG ATA AGT GCC GTA ATA TTG ACG GAA ATT TTA TCC TGA ATC
54	4[657]-0[644]	ACC ACC AGA GCC GCC ACC ACC CTC AGA GCA GAA CGC GCC TGT
55	4[993]-0[980]	CCA TCA CGC AAA TTC TTT ACA AAC AAT TTT TTC AAA TAT ATT
56	4[1329]-0[1316]	GGA ACC CTA AAG GGT GAT TGC TTT GAA TAA AAG AAG ATG ATG
57	5[336]-3[349]	TGA ATT ATC ACC GTG ATT AGC GGG GTT TAC GGC TAC AGA GGC
58	5[672]-3[685]	GAT AGC CCT AAA ACA TGG CTA TTA GTC TGC TGA CCT TCA TCA
59	5[1008]-3[1021]	AAA TCC TTT GCC CGA GTG AGG CCA CCG AAT CGG CCA ACG CGC
60	0[55]-2[42]	AAG CCC AAT AAT AAA GTA CCT TTA ATT GTT AAT TCG AGC TTC
61	3[980]-1[993]	AAA CCT GTC GTG CCC GTC GGT GGT GCC AGT AAT GGG ATA GGT
62	3[1316]-1[1329]	GGG CGA TGG CCC ACT TTC TCC GTG GTG AGA ACG GAT AAC CTC
63	4[363]-0[350]	AAG AGA AGG ATT AGC ACC GAC TTG AGC CGC CTT AAA TCA AGA
64	4[699]-0[686]	GAC AAT ATT TTT GAA TCG CCA TTA AAA ATT CTG TCC AGA CGA
65	4[1035]-0[1022]	GTG TTT TTA TAA TCA ACG TTA TTA ATT TCT GAT GCA AAT CCA
66	5[42]-3[55]	GCA GAT AGC CGA ACC TTT CCA GAC GTT ACG GAG TGA GAA TAG
67	5[378]-3[391]	GCC AGC AAA ATC ACT GAA AGT ATT AAG AAA GTT TCC ATT AAA
68	5[714]-3[727]	CCA GCA GAA GAT AAA CCT GAA AGC GTA AGG CCG TTT TCA CGG
69	5[1050]-3[1063]	ACA TTA TCA TTT TGC AGG AAC GGT ACG CCA GGG TGG TTT TTC
70	0[97]-2[84]	TTG AGC GCT AAT ATG ATG GCT TAG AGC TAA AAG ATT AAG AGG
71	3[1022]-1[1035]	GGG GAG AGG CGG TTT GGT CTG GTC AGC ACC GTG CAT CTG CCA
72	4[69]-0[56]	TAA AGT TTT GTC GTA AAG TTA CCA GAA GAC AAG AAT TGA GTT
73	4[405]-0[392]	ATT ATT CTG AAA CAC AGT AGC ACC ATT AGA GGC GTT TTA GCG
74	4[741]-0[728]	ATA GAA CCC TTC TGA ACA GAG GTG AGG CCA GTA ATA AGA GAA
75	4[1077]-0[1064]	AAA GGG ATT TTA GAC GGA ACA AAG AAA CTA ACC TCC GGC TTA
76	5[84]-3[97]	GCA ATA ATA ACG GAG ACA GCC CTC ATA GAT AAT TTT TTC ACG
77	5[420]-3[433]	GGA AAC GTC ACC AAT TAA TGC CCC CTG CGG CAC CAA CCT AAA
78	5[756]-3[769]	CCG CCT GCA ACA GTA ATA AAA GGG ACA TTG CCT GTT CTT CGC
79	5[1092]-3[1105]	GAA TTA TCA TCA TAA GAG CGG GAG CTA AAT TGC CCT TCA CCG
80	0[139]-2[126]	CGG GAG AAT TAA CTA CAT GTT TTA AAT ATT ACC CTG ACT ATT
81	3[1064]-1[1077]	TTT TCA CCA GTG AGC ATA ACG GAA CGT GTC AGG AAG ATC GCA
82	4[111]-0[98]	TGT AGC ATT CCA CAA TAC CCA AAA GAA CAA GTC AGA GGG TAA
83	4[447]-0[434]	GCC CGT ATA AAC AGT GAA ACC ATC GAT AGC AAA TCA GAT ATA
84	4[783]-0[770]	GTC ACA CGA CCA GTG CCA CGC TGA GAG CTA ACA ACG CCA ACA
85	4[1119]-0[1106]	TCC TCG TTA GAA TCT TCC TGA TTA TCA GTA GTG AAT TTA TCA
86	5[126]-3[139]	CTC CTT ATT ACG CAT TTC GTC ACC AGT AAA GGA GCC TTT AAT
87	5[462]-3[475]	AGT AGC GAC AGA ATT AAC GGG GTC AGT GCA CTC ATC TTT GAC
88	5[798]-3[811]	AAA TCT AAA GCA TCT GGA TTA TTT ACA TCC CCG GGT ACC GAG
89	5[1134]-3[1147]	TCA ATA TAA TCC TGT GCT TTG ACG AGC AGT CCA CGC TGG TTT

90	0[181]-2[168]	CTT TAC AGA GAG AAT CAT TCC ATA TAA CAG TTC AGA AAA CGA
91	3[1106]-1[1119]	CCT GGC CCT GAG AGT TTT CGT CTC GTC GGG TGC CGG AAA CCA
92	4[153]-0[140]	ACC GTA ACA CTG AGG TAT GTT AGC AAA CGG AAG CGC ATT AGA
93	4[489]-0[476]	CTG GTA ATA AGT TTC AAG TTT GCC TTT ATT TTA TTT TCA TCG
94	4[825]-0[812]	TCA ATC GTC TGA AAA CCT TGC TGA ACC TGC CAA CGC TCA ACA
95	4[1161]-0[1148]	GCG CGT ACT ATG GTA TTG TTT GGA TTA TAA ACA TAG CGA TAG
96	5[168]-3[181]	CAT AAA GGT GGC AAG GAT AGC AAG CCC AGT GAA TTT CTT AAA
97	5[504]-3[517]	CGC GTT TTC ATC GGA CAT GGC TTT TGA TAA GTA CAA CGG AGA
98	5[840]-3[853]	TCA ATC AAT ATC TGG CTC ATG GAA ATA CTT TCC TGT GTG AAA
99	5[1176]-3[1189]	GAA GGG TTA GAA CCC CCG CCG CGC TTA AGG TGG TTC CGA AAT
100	0[223]-2[210]	AGA AAC GAT TTT TTG ATT TAG TTT GAC CTC GTC ATA AAT ATT
101	3[1148]-1[1161]	GCC CCA GCA GGC GAG TTA AAC GAT GCT GCG CAA CTG TTG GGA
102	4[195]-0[182]	ACC CTC ATT TTC AGC ATA TAA AAG AAA CAT GAA AAT AGC AGC
103	4[531]-0[518]	CCA GTA AGC GTC ATC ATT TTC GGT CAT ATA TTA AAC CAA GTA
104	4[867]-0[854]	GCA ACA GGA AAA ACG TCA GTT GGC AAA TGT TTA GTA TCA TAT
105	4[1203]-0[1190]	GCG TAA CCA CCA CAT ACC ATA TCA AAA TGT AAA TCG TCG CTA
106	5[210]-3[223]	GAA TAA GTT TAT TTC CTC AGA ACC GCC AAT GAC AAC AAC CAT
107	5[546]-3[559]	GTT TGC CAT CTT TTG GAA AGC GCA GTC TGA AAT CCG CGA CCT
108	5[882]-3[895]	AAT TGA GGA AGG TTA TCC AGA ACA ATA TAT ACG AGC CGG AAG
109	5[1218]-3[1231]	ACA GAA ATA AAG AAG CGC TGG CAA GTG TTA GCC CGA GAT AGG
110	0[265]-2[252]	GTT ACA AAA TAA ACA CCT GTT TAG CTA TAT AGT AAA ATG TTT
111	3[1190]-1[1203]	CGG CAA AAT CCC TTT AAA AAA AGC CGC ATT ACG CCA GCT GGC
112	4[237]-0[224]	CTC AGA ACC GCC ACT GTC ACA ATC AAT ATC CCA ATC CAA ATA
113	4[573]-0[560]	ATT AAA GCC AGA ATC ATA ATC AAA ATC AAA TCA ATA ATC GGC
114	4[909]-0[896]	GCC TTG CTG GTA ATA TCT AAA ATA TCT TAA TAA GAA TAA ACA
115	4[1245]-0[1232]	GAG CGG GCG CTA GGA TTG CGT AGA TTT TCA GTA CAT AAA TCA
116	5[252]-3[265]	TTT ACC AGC GCC AAC GTA CTC AGG AGG TGC TGA GGC TTG CAG
117	5[588]-3[601]	ACC ACC GGA ACC GCC TTG ATA TTC ACA AGC AGA CGG TCA ATC
118	5[924]-3[937]	ACT AAT AGA TTA GAT GCC TGA GTA GAA GTG AGT GAG CTA ACT
119	5[1260]-3[1273]	GAT GAA TAT ACA GTG CGA GAA AGG AAG GAG TCC ACT ATT AAA
120	0[307]-2[294]	TTA CCA ACG CTA ACG TGG CAT CAA TTC TTA GCG AGA GGC TTT
121	1[70]-5[83]	AGG TCA TTT TTG CGC AGA GAG ATA ACC CGA AAC CGA GGA AAC
122	1[406]-5[419]	TTG TAC CAA AAA CAT ATT CTA AGA ACG CCC ATT AGC AAG GCC
123	1[742]-5[755]	ATA ATC AGA AAA GCA GGC ATT TTC GAG CGG TCA GTA TTA ACA
124	1[1078]-5[1091]	CTC CAG CCA GCT TTA GAG ACT ACC TTT TCA CCA GAA GGA GCG
125	2[167]-4[154]	GAA TGA CCA TAA ATA GCT TGC TTT CGA GAT AGG AAC CCA TGT
126	2[503]-4[490]	ACG TTG GGA AGA AAC CAA GCG CGA AAC AGA TAC AGG AGT GTA
127	2[839]-4[826]	TTT GCT CGT CAT AAC ATG GTC ATA GCT GCT ACA TTT TGA CGC
128	2[1175]-4[1162]	TGA TGA AGG GTA AAA AAT CCT GTT TGA TTG CGC CGC TAC AGG
129	3[224]-1[237]	CGC CCA CGC ATA ACC CAA TAC TGC GGA AAT TAG ATA CAT TTC
130	3[560]-1[573]	GCT CCA TGT TAC TTT TAA TTT CAA CTT TAG TCA AAT CAC CAT
131	1[112]-5[125]	AAT GCT GTA GCT CAG AAC ACC CTG AAC ATG GCA TGA TTA AGA
132	1[448]-5[461]	AAG CCT TTA TTT CAG CGC CCA ATA GCA AGC AGC ACC GTA ATC
133	1[784]-5[797]	ATA TTT AAA TTG TAA GAA TCG CCA TAT TCA GCA GCA AAT GAA
134	1[1120]-5[1133]	GGC AAA GCG CCA TTC TGA GAA GAG TCA AAT GAT GGC AAT TCA
135	2[209]-4[196]	CAT TGA ATC CCC CTT AGT TGC GCC GAC ACC CTC AGA GCC ACC
136	2[545]-4[532]	ACC TTA TGC GAT TTT GAT AAA TTG TGT CCT GAA TTT ACC GTT
137	2[881]-4[868]	CGG GTC ACT GTT GCC AAT TCC ACA CAA CTA CCG CCA GCC ATT
138	2[1217]-4[1204]	ATA AAA AAA TCC CGA TAA ATC AAA AGA AAG CGG TCA CGC TGC
139	3[266]-1[279]	GGA GTT AAA GGC CGT GCC AGA GGG GGT AAT TTT CAT TTG GGG
140	3[602]-1[615]	ATA AGG GAA CCG AAC GAG AAA CAC CAG ATA AAT TAA TGC CGG
141	1[154]-5[167]	GGT GTC TGG AAG TTT AAC ATA AAA ACA GGT AGA AAA TAC ATA
142	1[490]-5[503]	CAT ATA TTT TAA ATA ACA AGC AAG CCG TGC GTC AGA CTG TAG
143	1[826]-5[839]	TAA ATT TTT GTT AAC TTA CCA GTA TAA ACA AAT ATC AAA CCC
144	1[1162]-5[1175]	AGG GCG ATC GGT GCC TTA GAA TCC TTG AAC TTC TGA ATA ATG
145	2[251]-4[238]	AGA CTG GAT AGC GTC GAT ATA TTC GGT CTT AGT ACC GCC ACC
146	2[587]-4[574]	GGG CTT GAG ATG GTA GCC GGA ACG AGG CAC AAA TAA ATC CTC
147	2[923]-4[910]	CGC TTT CGC ACT CAC CTG GGG TGC CTA AAA CTC AAA CTA TCG
148	2[1259]-4[1246]	TGC TCA TTT GCC GCC AGT TTG GAA CAA GGA AGA AAG CGA AAG
149	3[308]-1[321]	GAA AGA CAG CAT CGG ATA AAA ACC AAA AAC TAA TAG TAG TAG
150	3[644]-1[657]	ACG GTG TAC AGA CCA CGT AAC AAA GCT GAA AGG CTA TCA GGT
151	1[196]-5[209]	TCT GCG AAC GAG TAG TTT AAC GTC AAA AGC AAA GAC ACC ACG
152	1[532]-5[545]	ATT CAA AAG GGT GAA TTC CAA GAA CGG GGC CCC CTT ATT AGC
153	1[868]-5[881]	GCC ATC AAA AAT AAA CTA GAA AAA GCC TCA ACA GTT GAA AGG
154	1[1204]-5[1217]	GAA AGG GGG ATG TGA TAA CCT TGC TTC TTA TTT GCA CGT AAA
155	2[293]-4[280]	TGC AAA AGA AGT TTC TTT TGC GGG ATC GTA AGT ATA GCC CGG
156	2[629]-4[616]	AAG GCT TGC CCT GAC TGA CCA ACT TTG AGG AGG TTG AGG CAG
157	2[965]-4[952]	CTT ACG GCT GGA GGT GCG CTC ACT GCC CTA CTT CTT TGA TTA
158	2[1301]-4[1288]	CGG AAA AAG AGA CGA CGT CAA AGG GCG AAG CTT GAC GGG GAA
159	3[350]-1[363]	TTT GAG GAC TAA AGT AGT AAG AGC AAC AAG GCA AAG AAT TAG
160	3[686]-1[699]	AGA GTA ATG CTG CGT CTG TGG TCT TGA CAG AAT CGA TGA ACG
161	1[238]-5[251]	GCA AAT GGT CAA TAA GCC ATA TTA TTT AGA AAA TTC ATA TGG
162	1[574]-5[587]	CAA TAT GAT ATT CAA GCA TGT AGA AAC CCC GGA ACC AGA GCC
163	1[910]-5[923]	TTC ATC AAC ATT AAT AAA TAA GGC GTT ATA GGA GCA CTA ACA
164	1[1246]-5[1259]	AGG GTT TTC CCA GTT TTT TTA ATG GAA ACA GGT TTA ACG TCA
165	2[335]-4[322]	GTT TAC CAG ACG ACG AAC GAG GGT AGC ATG CTC AGT ACC AGG
166	2[671]-4[658]	CAT TAC CCA AAT CAA GGC GCA TAG GCT GTT AAT GCC AGA ACC
167	2[1007]-4[994]	GCC AAC GGC AGC ACA GCT GCA TTA ATG AGT AAA AGA GTC TGT
168	3[56]-1[69]	AAA GGA ACA ACT AAT CAA ATA TCG CGT TCT CCT TTT GAT AAG
169	3[392]-1[405]	CGG GTA AAA TAC GTC AAC TAA TGC AGA TTA AAG CTA AAT CGG
170	3[728]-1[741]	TCA TAC CGG GGG TTG CAT CAG ACG ATC CAT GTA CCC CGG TTG
171	1[280]-5[293]	CGC GAG CTG AAA AGG AGC GTC TTT CCA GCA TTC AAC CGA TTG
172	1[616]-5[629]	AGA GGG TAG CTA TTT AAG TCC TGA ACA ACC ACC CTC AGA ACC
173	1[952]-5[965]	CTC CGT GGG AAC AAC TTC TGA CCT AAA TAT ACA TTT GAG GAT
174	1[1288]-5[1301]	CAA GCT TTC AGA GGA AAA CAA AAT TAA TCA TCG GGA GAA ACA
175	2[377]-4[364]	GGA ATT ACG AGG CAA CTT TTT CAT GAG GGG CTG AGA CTC CTC
176	2[713]-4[700]	GCG CGC CTG TGC ACG CCA GAA TGC GGC GGA ATA CGT GGC ACA
177	2[1049]-4[1036]	CGT CAG CGT GGT GCT GCG TAT TGG GCG CCA GAA TCC TGA GAA
178	3[98]-1[111]	TTG AAA ATC TCC AAA GCG GAT TGC ATC ATA ATT GCT GAA TAT
179	3[434]-1[447]	ACG AAA GAG GCA AAA GGT AGA AAG ATT CTA CTT TTG CGG GAG
180	3[770]-1[783]	GTC CGT GAG CCT CCC AGA TGC CGG GTT AGA TTG TAT AAG CAA
181	1[322]-5[335]	CAT TAA CAT CCA ATG CAC CCA GCT ACA ATT ATT CAT TAA AGG
182	1[658]-5[671]	CAT TGC CTG AGA GTA TGT TCA GCT AAT GCC GCC ACG CGA ACT
183	1[994]-5[1007]	CAC GTT GGT GTA GAA ACG CGA GAA AAC TCG ACA ACT CGT ATT
184	2[83]-4[70]	AAG CCC GAA AGA CTA GGA ATT GCG AAT ATT AGC GTA ACG ATC

185	2[419]-4[406]	AGG AAT ACC ACA TTA ATG CCA CTA CGA ACT ATT TCG GAA CCT
186	2[755]-4[742]	TGC CGG TGC CCC CTT CTG CCA GCA CGC GTC TGG CCA ACA GAG
187	2[1091]-4[1078]	CCA GAG CAC ATC CTA CGG GCA ACA GCT GAC AGG AGG CCG ATT
188	3[140]-1[153]	TGT ATC GGT TTA TCC AAA AAT CAG GTC TTG CAA CTA AAG TAC
189	3[476]-1[489]	CCC CAG CGA TTA TAA ATC TAC GTT AAT AAT TTT TAG AAC CCT
190	3[812]-1[825]	CTC GAA TTC GTA ATA CAT CCC TTA CAC TGT TAA AAT TCG CAT
191	1[364]-5[377]	CAA AAT TAA GCA ATC GGG AGG TTT TGA AAT TTG GGA ATT AGA
192	1[700]-5[713]	GTA ATC GTA AAA CTA AAA GGT AAA GTA ATA CCG AAC GAA CCA
193	1[1036]-5[1049]	GTT TGA GGG GAC GAA CTA TAT GTA AAT GTA AAA GTT TGA GTA
194	2[125]-4[112]	ATA GTC AGA AGC AAA AAA AAG GCT CCA ACA AAC TAC AAC GCC
195	2[461]-4[448]	AAC AAC ATT ATT ACA GAA TAC ACT AAA ACC TTG AGT AAC AGT
196	2[797]-4[784]	ATC GTT AAC GGC ATT CAC AGT TGA GGA TTG GCA GAT TCA CCA
197	2[1133]-4[1120]	AAA CGC GGT CCG TTA GTT GCA GCA AGC GCG TAT AAC GTG CTT
198	3[182]-1[195]	CAG CTT GAT ACC GAC AAA TGC TTT AAA CAG TTG ATT CCC AAT
199	3[518]-1[531]	TTT GTA TCA TCG CCT AAG AAC TGG CTC AAT GTG TAG GTA AAG
200	3[854]-1[867]	TTG TTA TCC GCT CAC CTG CGG CTG GTA ATA ACC AAT AGG AAC

Table S2 Estimated cost of materials for gradient mixer

Description	Purpose	Quantity	Price @	Total price
ELEGOO UNO R3 Board ATmega328P	Microcontroller	1	\$ 13.99	\$ 13.99
6 VDC Mini motor	Motor	1	\$ 0.75	\$ 0.75
SG92R Mini servo	Servo	1	\$ 4.75	\$ 4.75
Qunqi L298N Motor Drive Controller Board	Motor Controller	1	\$ 6.69	\$ 6.69
OVERTURE PLA Filament	3D Print Material	300 gr	\$ 18.99 /kg	\$ 6.00
			**Total Price**	\$ 32.18

S3. Protocols

Reagents

Concentration gradient

- 50× TAE buffer, pH 8.3 (VWR Life Science, cat. no. 75800-940)
- Magnesium chloride hexahydrate (Sigma-Aldrich, cat. no. M9272)
- Glycerol (VWR Life Science, cat. no. BDH1172)
- Sucrose (Sigma-Aldrich, product no. S0389)

DNA origami folding

- 50× TAE buffer, pH 8.3 (VWR Life Science, cat. no. 75800-940)
- Magnesium chloride hexahydrate (Sigma-Aldrich, cat. no. M9272)
- Staple strands (Integrated DNA Technique Inc.)
- ssDNA scaffold p8064 (Tilibit Nanosystem, product no. M1-51)

Agarose Gel Electrophoresis

- SYBR gold DNA gel stain (Thermo Fisher Scientific, cat no. S11494)
- DNA gel loading dye (Thermo Fisher Scientific, cat no. R0631)
- 1 kb DNA ladder (Gold Biotechnology Inc., cat. no. D010-500)
- Agarose tablets (EURx, cat. no. E0305-01)

Equipments

General

- P2L Eppendrof pipette (Gilson, SKU FA10001M)
- P10L Eppendrof pipette (Gilson, SKU FA10002M)
- P200L Eppendrof pipette (Gilson, SKU FA10005M)
- P1000L Eppendrof pipette (Gilson, SKU FA10006M)
- EXPERT tips E200 Tipack (Gilson, SKU F1733002)
- EXPERT Tips E1000 XL Tipack (Gilson, SKU F1735002)
- Milli-Q® Direct Water Purification System (Milipore Sigma, cat. no. ZR0Q016WW)

Density gradient preparation

- EV3 LEGO Mindstorm (LEGO, item no. 31313)
- Ultimaker 3 3D printer (part no. 9671)
- PLA 3D printing materials
- 1 mL syringe (Becton Dickinson, cat. no. 309628)

DNA origami preparation

- Thermocycler, MiniAmp^{TM} Thermal Cycler REX (Thermo Fisher Scientific, cat No. A38080)
- PCR tubes (USA Scientific, cat. no. 1402-8120)
- 100 kD amicon filter (Milipore, cat. no. UFC510024)
- Benchmark MC-12 High Speed Microcentrifuge (Marshall Scientific, item no. C1612)

Rate-zonal centrifugation

- Beckman TLS 55 swinging bucket rotor (Beckman Coulter, part no. 346936)
- Polycarbonate centrifuge tube (Beckman Coulter, part no. 343778)
- Optima TLX ultracentrifuge (Beckman Coulter, part no. 361545)
- Longneck gel loading tips (Fisher Scientific, cat. no. 02-707-81)
- Safe Imager 2.0 Blue Light transilluminators (Invitrogen, cat. no. G6600)

Gel electrophoresis

- 250 mL Erlenmeyer flask (Karter Scientific, part no. 214U2)
- External gel cast (Thermo Fisher Scientific, cat. no. B2-CST)
- Owl easyCast^{TM} B2 Mini Gel Electrophoresis Systems (Thermo Fisher Scientific, cat. no. B2-BP)
- Bio-Rad Molecular Imager Gel Doc XR+ (Bio-Rad, part no. 1708195EDU)

Atomic force microscopy

- MultiMode 8-HR AFM (Bruker)
- ScanAsyst-Fluid+probes cantilever (Bruker)
- Glass probe holder MTFML-V2 (Bruker)
- V1 AFM mica discs, 10mm (Ted Pella Inc., product no. 50-10)
- AFM/STM Metal Specimen Discs, 12mm (Ted Pella Inc., product no. 16208)

Protocols

DNA origami preparation

1 | Referring to the sequences of staple strands (Table S1), group the staple strands into the following categories.

Category	Staple Strands
Left ON	staple 1–2
Right ON	staple 3–4
Left OFF	staple 5–8
Right OFF	staple 9–12
Short 1 2	staple 13–14
Short 3 4	staple 15–16
Core 6-hb	the rest of the staples

2 | To create the 6-hb monomer mixture, combine 30 nm of the ssDNA p8064 scaffold strands and 300 nm each of the staple strands from Left OFF, Right OFF, and Core 6-hb. The strand mix should be added in 1× TAE 12.5 mM $MgCl_2$ (final concentration).

3 | To create the 6-hb dimer mixture, two types of precursor monomer mixtures, *left* and *right*, need to be mixed in separate tubes. The *left* precursor monomers have complementary sequences with the *right* precursor monomer, enabling them to form a dimer. The *left* precursor monomer contains 30 nm of the ssDNA p8064 scaffold strands and 300 nm each of the staple strands in Left ON, Right OFF, Short 1 2, and Core 6-hb. The *right* precursor monomer contains 0 nm of the ssDNA p8064 scaffold strands and 300 nm each of the staple strands in Right ON, Left OFF, Short 3 4, and Core 6-hb. All origami mixture is in 1× TAE 12.5 mM $MgCl_2$ (final concentration).

4 | Anneal the 6-hb monomer, *left* precursor monomer and *right* precursor monomer in separate PCR tubes using a thermocycler with a thermal gradient that starts at 90°C, gradually cools to 30°C over 1.5 hours, and ends with incubation at 4°C. The 6-hb monomer is formed after this annealing process. The 6-hb dimer needs an additional step after the *left* and *right* precursor monomers are formed.

5 | Filter excess staple from the *left* and *right* monomer separately using 100 kD Amicon filtration on a centrifuge. Both precursor monomers should be filtered at 4,500 g for 5 minutes twice to ensure optimal staple filtration while maintaining the integrity of the DNA origami structure. After filtering the mixture twice, collect the DNA origami by flipping the amicon filter on a new tube and centrifuging them at 1,000 g for 2 minutes.
CRITICAL STEP Filtration of excess staples from precursor monomers is important to ensure that the leftover staple from one precursor does not bind to the activated site of the other precursor, preventing dimerization by binding to the activated site.

6 | Combine the *left* and *right* precursor monomer after filtration of excess staples in step 5. The combined mixture is stored at 4°C to dimerize overnight. The 6-hb dimer is formed after this process.

7 | Once the 6-hb monomers and dimers are formed, perform an AGE analysis to verify the formation of both origami. The monomer shows one band, while the dimer shows two bands. The two bands in the dimer lane show one band at the same level as the monomer (from the precursor monomer that fails to dimerize) and another band slightly below the monomer (indicating the dimer).

Preparation of density gradient

8 | Assemble the LEGO gradient machine following the instructions in Figure S1. The LEGO parts needed to build the LEGO gradient mixer can be found in the EV3 LEGO Mindstorm pack.

9 | 3D print the centrifuge tube holders file with PLA materials. The software used to produce the .gcode needed for 3D printing is Ultimaker Cura and the 3D printer used to print the centrifuge tube holder is Ultimaker 3. Any other 3D printer can also be used to print the centrifuge tube holder. Once the centrifuge tube holder is printed, attach the holder to the spinning motor (Figure 1b) of the LEGO assembly. The full assembly of the LEGO gradient mixer is shown in Figure 1.

10 | Prepare several different concentrations of glycerol (or sucrose, depending on the viscous agent used). A common gradient used in the experiment is a 15%–45% (v/v) gradient.

11 | Fill the centrifuge tube with 300 μL of the 45% glycerol using a pipette. Make sure that the 45% glycerol is resting on the bottom of the tube and that there is no bubble present. After 45% glycerol, lay 300 μL of 15% glycerol on top of the 45% glycerol in the centrifuge tube using a syringe to ensure minimal surface disturbance in the 45% glycerol layer. The border between the 15% and 45% glycerol should be visible.

12 | Load the centrifuge tube filled with glycerol into the designated holder in the LEGO gradient mixer. Run the LEGO__gradient__mixer__protocol.ev3 protocol found in the Supporting Information Repository, EV3-protocols folder. The protocol will tilt the 90 °glycerol-filled tube 90°to a horizontal position and spin the tubes for one minute to mix the two different concentrations of glycerol. After spinning the tubes, the LEGO gradient mixer will return the tubes to its initial position. Now, a faded boundary and a smooth gradient between the 15% and 45% glycerol can be observed.

Rate Zonal Centrifugation and Fractionation

13 | Prepare the sample for RZC by mixing it with glycerol to a 10% (v/v) final concentration. Adding glycerol to the sample ensures that the sample sits on the top surface of the glycerol gradient and eases the entry of the sample into the glycerol gradient during RZC.

14 | Carefully load 50-100 μL of the sample (10% glycerol) on top of the gradient using a pipette, making sure the sample does not penetrate the gradient. Most of the sample should rest on the surface of the gradient.

15 | Insert the centrifuge tubes containing the sample and gradient into a swinging-bucket rotor (Beckman TLS 55).
CRITICAL STEP A swinging-bucket rotor should be used instead of a fixed-angle or vertical rotor because the glycerol gradient used is positioned from top to bottom. To prevent the sample from pelleting on the side and to

ensure that the sample travels down the gradient, a swinging bucket rotor is needed.

16 | Centrifuge the rotor at 50,000 rpm (∼150,000 g) for 1.5 hours at 4°C in the Optima TLX ultracentrifuge. For different origami, the RPM and the duration of centrifugation need to be optimized.

17 | Once the centrifugation is complete, collect 16 to 18 equal volume fractions of the gradient containing the sample. Longneck gel loading tips can be used with a pipette to fractionate the gradient from bottom to top. For a manual method such as this, fractionation from bottom to top is recommended because it ensures a more organized way to control the position of the tips. Other methods of fractionation using liquid handling instruments are preferred to achieve a more consistent RZC result.

Agarose Gel Electrophoresis

18 | After the sample has been fractionated, take 10 µL from each fraction and mix it with 1 µL of 20× SYBR gold and 1 µL of loading dye. Leave it to incubate at room temperature for 30 min.

19 | Cast a 1% agarose gel with enough lanes to accommodate the number of fractions being analyzed.

20 | When the gel is ready to use, put the gel into its electrophoresis chamber and fill the chamber with 1× TAE 12.5 mM $MgCl_2$ buffer until the gel is submerged.

21 | Load 1 kb DNA ladder and all fractions that have been labeled with SYBR gold (step 18) into their respective lanes on the gel. Start the electrophoresis using a 75 V power supply for 1.5 hours.

22 | After the gel electrophoresis is done, image the gel using Bio-Rad Molecular Imager Gel Doc XR+. Set the setting to image nucleic acids with SYBR Gold label. The bands in the gel show the staple strands near the top fraction lanes and the origami somewhere in the middle to bottom fractions. If the bands are not immediately clear, post-staining the gel in 1× SYBR Gold for 15–30 minutes will increase the band intensity.

23 | Combine the fractions containing the origami (based on the AGE result) into a single tube consisting of the purified origami.

AFM imaging

24 | Turn on the MultiMode 8 controller and AFM to warm up the laser.

25 | Mount the 10 mm V1 mica discs onto the AFM/STM 12 mm metal specimen discs and cleave the mica sheet with a tape to an atomically flat surface for AFM imaging.

26 | Dilute the fractionated sample by 100× with 1× TAE 12.5 mM $MgCl_2$. Depending on the concentration of the purified sample, the dilution factor may need to be adjusted.

27 | Pipette 30 µL of the diluted sample onto the freshly cleaved mica. Sometimes, a larger sample is added to cover the mica surface. Place the mica puck containing the sample inside the AFM.

28 | Mount the scanAsyst-Fluid+ probes cantilever to the glass probe holder MTFML-V2 with a tweezer and insert them into the AFM.

29 | Lower the cantilever until it touches the sample. Adjust the laser position and mirror to reach a gain of at least 6.00.

30 | Calibrate the zero position for the vertical and horizontal position and engage the cantilever to begin scanning. The scanned images are flattened and the length of the 6-hb origami(s) is measured to determine the presence of monomers (∼500 nm) and dimers (∼1000 nm).

S4. Supporting Information Repository

The instructions for assembling the LEGO gradient mixer and all the data supporting this study are provided at https://zenodo.org/badge/latestdoi/446256259 .

Movie S1 **Protocol video instruction to use the LEGO gradient mixer.** The recording is a demonstration for the creation of a sucrose gradient with LEGO machine and RZC purification of the sample, 140 nm green fluorescent beads. Protocol performed in the recording: layering the sucrose on the centrifuge tube, creating the gradient with the LEGO machine, loading the sample to the gradient, and centrifugation to purify the sample.
https://github.com/jsentosa3/LEGO-gradient-maker/tree/main/Recording-protocols
