# Supplementary material for: Gradient-mixing LEGO robots for purifying DNA origami nanostructures of multiple components by rate-zonal centrifugation
Source: PLoS One. 2023 Jul 19;18(7):e0283134. doi: 10.1371/journal.pone.0283134 (PMC10355445; doi:10.1371/journal.pone.0283134)
Supplement: Attachment — Submitted filename: Cover_Letter -- Response_to_Reviewers -- combined.pdf [file pone.0283134.s002.pdf]

Rizal F. Hariadi
Assistant Professor

Department of Physics
Biodesign Institute
Arizona State University

+1.626.376.8638
rhariadi@asu.edu

October 31, 2022

Dear Editors,

We respectfully submit the revised version of our article "Gradient-mixing LEGO robots for purifying DNA origami nanostructures of multiple components by rate-zonal centrifugation"). We appreciate all reviewers' support, as well as thoughtful comments. The manuscript has been revised, a new supplementary movie has been prepared, and new sentences have been added to address all the concerns of the reviewer. The feedback from the editors and referees significantly improved the original submission.

Start of Responses to Journal Requirements

1. *Please ensure that your manuscript meets PLOS ONE's style requirements, including those for file naming. The PLOS ONE style templates can be found at*

   https://journals.plos.org/plosone/s/file?id=wjVg/PLOSOne_formatting_sample_main_body.pdf and https://journals.plos.org/plosone/s/file?id=ba62/PLOSOne_formatting_sample_title_authors_affiliations.pdf

   The manuscript has been reformatted to be compliant with PLOS ONE's style requirements as described in this cover letter.

2. *Thank you for stating the following financial disclosure:*
   "The research in Hariadi lab was supported by the National Institutes of Health Director's New Innovator Award (1DP2AI144247), National Science Foundation SemiSynBio II (2027215), and Arizona Biomedical Research Consortium (ADHS17-00007401)."
   Please state what role the funders took in the study. If the funders had no role, please state: "The funders had no role in study design, data collection and analysis, decision to publish, or preparation of the manuscript." Please include this amended Role of Funder statement in your cover letter; we will change the online submission form on your behalf.
   If this statement is not correct you must amend it as needed.
   Please include this amended Role of Funder statement in your cover letter; we will change the online submission form on your behalf.

We have revised our financial disclosure statement to state that the funders had no role in the study.

> The research in Hariadi lab was supported by the National Institutes of Health Director's New Innovator Award (1DP2AI144247), National Science Foundation SemiSynBio II (2027215), and Arizona Biomedical Research Consortium (ADHS17-00007401). **The funders had no role in study design, data collection and analysis, decision to publish, or preparation of the manuscript**.

3. *Thank you for stating the following in the Acknowledgments Section of your manuscript: "The research in Hariadi lab was supported by the National Institutes of Health Director's New Innovator Award (1DP2AI144247), National Science Foundation SemiSynBio II (2027215), and Arizona Biomedical Research Consortium (ADHS17-00007401)."*
   We note that you have provided additional information within the Acknowledgments Section that is not currently declared in your Funding Statement. Please note that funding information should not appear in the Acknowledgments section or other areas of your manuscript. We will only publish funding information present in the Funding Statement section of the online submission form.
   Please remove any funding-related text from the manuscript and let us know how you would like to update your Funding Statement. Currently, your Funding Statement reads as follows: "The research in Hariadi lab was supported by the National Institutes of Health Director's New Innovator Award (1DP2AI144247), National Science Foundation SemiSynBio II (2027215), and Arizona Biomedical Research Consortium (ADHS17-00007401)." Please include your amended statements within your cover letter; we will change the online submission form on your behalf.

   We have removed the funding statement from the Acknowledgments section of the manuscript. The funding statement on the initial manuscript is accurate and in compliance with PLOS ONE's policy.

4. *We note that you have stated that you will provide repository information for your data at acceptance. Should your manuscript be accepted for publication, we will hold it until you provide the relevant accession numbers or DOIs necessary to access your data. If you wish to make changes to your Data Availability statement, please describe these changes in your cover letter and we will update your Data Availability statement to reflect the information you provide.*

   We have generated a DOI for our Supporting Information Repository in github using zenodo.com. The DOI is https://zenodo.org/badge/latestdoi/446256259

5. *Please amend the manuscript submission data (via Edit Submission) to include author Robert Rezvani.*

   Thank you for pointing the missing author information out. We have included Robert Rezvani in the author list on the submission portal.

6. *PLOS ONE now requires that authors provide the original uncropped and unadjusted images*

underlying all blot or gel results reported in a submission's figures or Supporting Information files. This policy and the journal's other requirements for blot/gel reporting and figure preparation are described in detail at https://journals.plos.org/plosone/s/figuresloc-blot-and-gel-reporting-requirements and https://journals.plos.org/plosone/s/figuresloc-preparing-figures-from-image-files. When you submit your revised manuscript, please ensure that your figures adhere fully to these guidelines and provide the original underlying images for all blot or gel data reported in your submission. See the following link for instructions on providing the original image data: https://journals.plos.org/plosone/s/figuresloc-original-images-for-blots-and-gels. In your cover letter, please note whether your blot/gel image data are in Supporting Information or posted at a public data repository, provide the repository URL if relevant, and provide specific details as to which raw blot/gel images, if any, are not available. Email us at plosone-plos.org if you have any questions.

We have uploaded the original/uncropped gel images in Figures 4B and G into a public data repository. The images and its information can be found in the Supporting Information Repository (https://github.com/ jsentosa3/LEGO-gradient-maker). by the name of SI_raw_images.pdf

7. *Please include captions for your Supporting Information files at the end of your manuscript, and update any in-text citations to match accordingly. Please see our Supporting Information guidelines for more information: http://journals.plos.org/plosone/s/supporting-information.*

   We revised the manuscript to include captions, with their titles, for all the Supplementary Figures and Sections in the Supporting Information of the revised manuscript.

8. *Please review your reference list to ensure that it is complete and correct. If you have cited papers that have been retracted, please include the rationale for doing so in the manuscript text, or remove these references and replace them with relevant current references. Any changes to the reference list should be mentioned in the rebuttal letter that accompanies your revised manuscript. If you need to cite a retracted article, indicate the article's retracted status in the References list and also include a citation and full reference for the retraction notice.*

   We have reviewed our references and we found no retracted papers. There were no changes to the Reference list.

End of Responses to Journal Requirements

Start of Responses to Additional Editor's comments

1. *In Fig. 1, please adjust the background to show black wires or modify the color of the wires; change a, b, . . . ,f to numbers, similar to Fig. 2,*

   We appreciate the suggestions for Figure 1. Figure 1 has been revised to make the black wires more pronounced by tracing the black wires with white lines on top of the black background. In Figure 1, we also changed the small letter labels to numbers, as suggested.

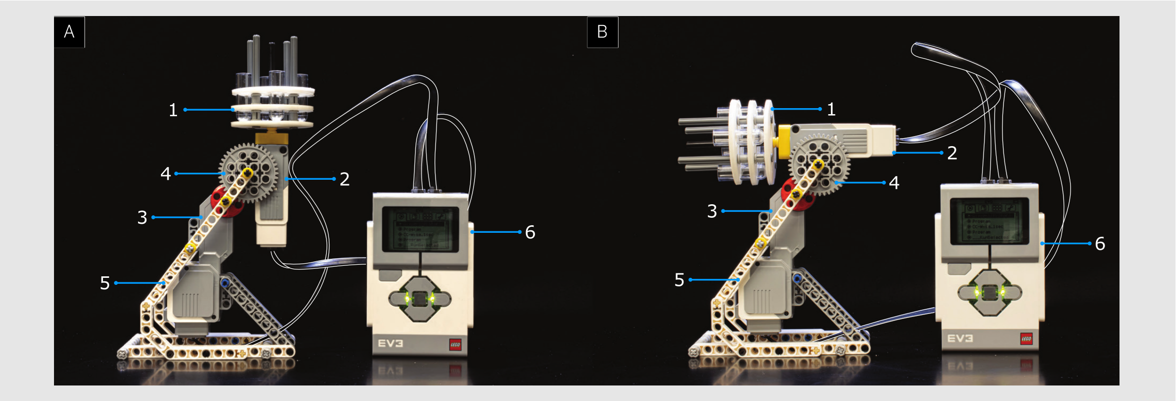

2. *Instead of building instruction, generate an animation for the assembly of the Gradient Mixer.*

   We have included a .gif animation for the assembly of the Gradient Mixer in the Supporting Information (https://github.com/jsentosa3/LEGO-gradient-maker/tree/main/S1_Fig-LEGO_Building_Instruction)

3. *The authors mentioned that Fig. 4 C and D demonstrated a significant difference between unpurified and purified samples. However, the quantitative analysis, such as surface roughness of background area in AFM images, will be more convincing than visual inspection.*

   We thank the editor for suggesting a more quantitative approach to demonstrate the difference after purification. AFM images of unpurified (left) and purified (right) monomers and their corresponding height distributions (gray histograms). We modeled the AFM pixel height distribution ($n(z)$) data as a sum of 3 Gaussian functions (black) corresponding to 3 groups, namely the mica surface (blue), the staple strands (red) and DNA origami nanostructures (green).

$$n(z) \propto P_{mica}\frac{1}{\sigma_{mica}}e^{-\frac{1}{2}\left(\frac{z-\mu_{mica}}{\sigma_{mica}}\right)^2} + P_{staple}\frac{1}{\sigma_{staple}}e^{-\frac{1}{2}\left(\frac{z-\mu_{staple}}{\sigma_{staple}}\right)^2} + P_{origami}\frac{1}{\sigma_{origami}}e^{-\frac{1}{2}\left(\frac{z-\mu_{origami}}{\sigma_{origami}}\right)^2} \quad (1)$$

   The cumulative distribution of the pixel heights was fitted to the cumulative distribution function (CDF) of Eq. 1 with the following fitting parameters

$$P_{mica} = \text{fraction of pixels with only mica surfaces}$$
$$P_{staple} = \text{fraction of pixels with staple strands}$$
$$P_{mica} = \text{fraction of pixels with DNA origami nanostructures}$$
$$\mu mica = \text{mean height of mica surfaces}$$
$$\mu staple = \text{mean height of staple strands}$$
$$\mu mica = \text{mean height of DNA origami nanostructures}$$
$$\sigma_{mica} = \text{standard deviation of the observed height of mica surfaces}$$
$$\sigma_{staple} = \text{standard deviation of the observed height of staple strands}$$
$$\sigma_{mica} = \text{standard deviation of the observed height of DNA origami nanostructures}$$

and with a constraint

$$P_{mica} + P_{staple} + P_{origami} = 1$$

Based on the fit, the inferred percentages of pixels corresponding to the staple strands (P_{staple}) are 62% in unpurified monomer and 29% in purified monomer. After purification, P_{staple} is reduced by 2.14×, consistent with the expectation that RZC removes the majority of excess staple strands and excess staple strands. Custom code for CDF analysis in `Mathematica` is available in Supporting Information Repository. The analysis was incorporated into Figure. 4, the following is the updated Figure. 4:

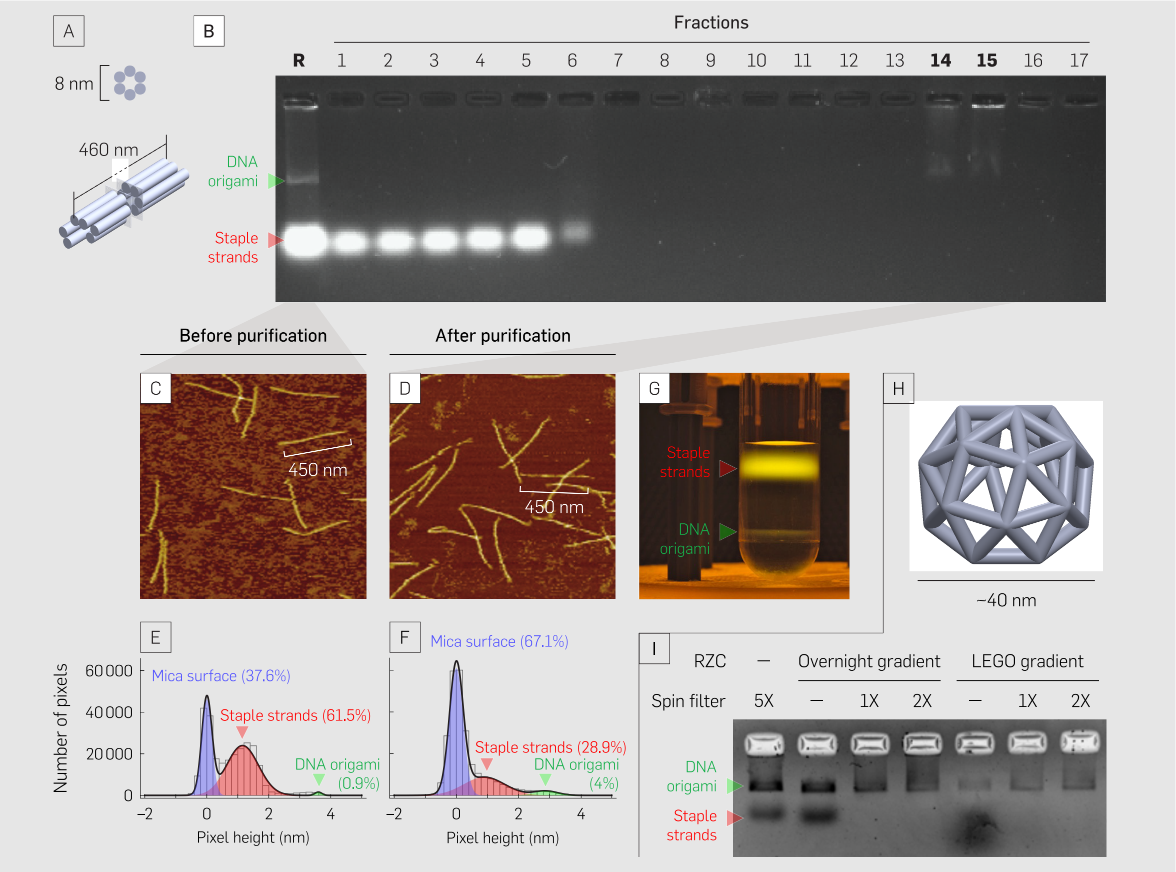

We also added the following sentences in the main text.

> ...Based on the fit, the inferred percentages of pixels corresponding to the staple strands (P_{staple}) are 62% in unpurified monomer and 29% in purified monomer. After purification, P_{staple} is reduced by 2.14×, consistent with the expectation that RZC removes the majority of excess staple strands and excess staple strands.

4. *Regarding blot/gel data: PLOS ONE now requires that submissions reporting blots or gels include original, uncropped blot/gel image data as a supplement or in a public repository. This is in addition to complying with our image preparation guidelines described at https://journals.plos.org/plosone/s/figuresloc-blot-and-gel-reporting-requirements. These requirements apply both to the main figures and to cropped blot/gel images included in Support-*

ing Information.

We have uploaded the original/uncropped gel images in Figures 4B and G into a public data repository. The images and its information can be found in the Supporting Information Repository (https://github.com/ jsentosa3/LEGO-gradient-maker). by the name of SI_raw_images.pdf

5. *On page 5, there is a typo "applicatios".*

   We thank the editor for catching this typo. We have corrected the typo on page 5.

6. *Please provide the sample size (total numbers of images or molecules) in the statistical analysis.*

   We thank the editor for catching that some of the measurements are not accompanied by the information about the sample size (N). We have included the number of images, samples, or molecules for each measurement throughout the revised manuscript.

End of Responses to Editorial Requests

Start of Responses to Referees

Reviewer #1: *The Jason et al. manuscript describes good equipment which can be well compact with the rate-zonal centrifugation in purifying DNA. The LEGO robots they made are very easily assembled and generate the critical point of purification (glycerol gradient) very high efficiency. Overall, this is a good method paper to make the new equipment. However, purifying DNA (RZC) is not new and well developed. Therefore, the insight point for this paper is to complete a smooth gradient of glycerol for RZC.*

We thank Reviewer 1 for the positive evaluation.

1. *The authors made this LEGO robot with many Lego pieces and wrote the control code. And the function of this LEGO robot is to flip 90 degrees and rotate for a particular time. There are a lot of simple motors which can spin the samples. What is the advantage of this machine?.*
   Thank you for your feedback. We agree that there are other simpler motors, such as stepper motors or servos that can serve as an adequate substitute for the LEGO robot. Our main purpose to use LEGO is due to the versatility of its components, which allows us to quickly produce a working prototype, and its standardized units, which increases the reproducibility of our experiment. To create the gradient mixer robot using the aforementioned substitutes will require us to also 3D print the scaffold and other components to hold the machine which added another layer of complexity in reproducing the experiment. Although we acknowledged that using LEGO is not the most cost-effective way to build the machine, it is sufficient to demonstrate the concept we introduced in the manuscript. The revised text reads as follows from line 156 in the revised review:

> ...The main components of the LEGO gradient mixer are a motor, a servo, and a custom holder for the tubes, all of which do not require a high degree of precision to be effective. Hence, the price to create such a LEGO gradient mixer can be reduced further by an order of magnitude (Table S2). To lower the cost further, such machines can be powered manually using some gear and scaffolding materials...

2. *In figure 5D, the difference between monomer and dimer is apparent, and there is a big gap between them. Why are there 37% of the monomer in the purified dimer products? I ask this because sometimes the purity of the target product is critical and 63% of the purity is not high. If the difference between monomer and dimer is similar in the agarose gel, you can make the purity 100%.*
We highly value your concern for the yield of purification. We acknowledged in our manuscript that purity is important in DNA origami applications and the current result from our manuscript have not demonstrate the necessary purity of product. When visualized with a blue light transilluminators, our SYBR gold-stained dimer clearly show the separation between the monomer and dimer (Fig. 5B-D). This led us to believe that the impurities in our dimer product happens after the purification step with RZC, the sample extraction step. We hypothesized that the impurities arises due to our crude method of extracting the sample after separation by RZC (fractination). We used pipettes equipped with long neck gel loading tips which either disturb the separated product (6-hb monomer and dimer), accidentally mixing it, or mechanically break the dimers, separating it into two monomers. This might explain the monomer that are present in the purified dimer AFM images. Improvement on extraction technique may produce more consistent and higher purity of the product.

> ...Although the purified dimer shows only 63±6% purity (Fig 5J), the observed monomer in the purified dimer likely results from the difficulty of extracting the dimer sample manually from the small gradient volume in which the experiment is conducted. Taking note that we used less than ideal equipment for the purification method, *i.e.* long neck gel loading tips, the yield could significantly be improved by upgrading or automating the fractionation technique. An improved and/or automated fractionation technique could produce more consistent and higher yield results and would therefore be integral to optimizing the effectiveness of RZC purification.

3. In figure 2, the authors used glycerol gradient but used sucrose in the movie.
Thank you for pointing this difference. We used different viscous solution to create the density gradients to demonstrate the LEGO gradient mixer versatility in handling different materials depending on the research need.

> ...Alternatively, sucrose can be used to substitute glycerol. A sucrose gradient created using the LEGO gradient mixer was able to separate the dimer with similar effectiveness to the glycerol gradient (see Figs. S3 for dimer separation using sucrose gradient).

4. Does vision only distinguish the gradient of different glycerol in Fig.3? Are there any measurable parameters to quantify the gradient? Are these results repeatable very well each time?.
We have improved on our previous inspection of the glycerol gradient by quantifying its gray value in a plot profile. We measured the gray value of a dyed glycerol gradient (Fig. 3) from

the top (clear) to bottom (blue). The gray values were then plotted over its distance. After normalizing the gray value into a scale of 0 to 1 and the distance to 1000 points, we did a reproducibility experiment comparing images of 20 glycerol gradients created using the same setting in the LEGO gradient mixer (15% glycerol top, 45% glycerol bottom, 60 sec spin time). The result showed that the glycerol gradients created by the LEGO gradient mixer were consistent. The standard deviation of the plot profile form the mean was measured at 7.1%, indicating similarities in the quality of the gradient produced.

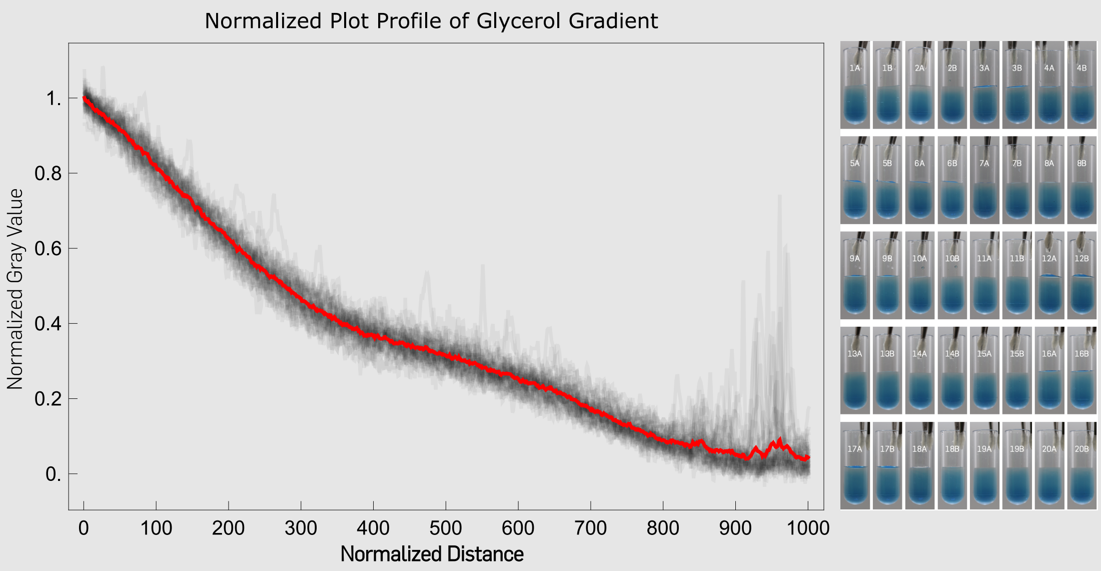

...The consistency of the gradient produced was tested by calculating the deviation from plot profiles of multiple gradients created by LEGO gradient mixer (Fig S3). The deviation was calculated at 7.1%, indicating that the gradient generated by LEGO LEGO gradient machine is consistent.

Reviewer #2: In this work, the authors made LEGO robots to prepare RZC for purifying DNA origami nano-structures. They tested three kinds of DNA origami structures with different shapes and strand ratios. The results look promising and show the effectiveness of the robot's gradient-mixing RZC. Although it is not a large-load work, it is interesting and inspires the perspective of nanostructure synthesis with robots. I am just curious how well the robot can be programmed.

We thank Reviewer 2 for his/her appreciation of our work.

End of Responses to Referees

Thank you for your careful evaluation,

Jason Sentosa, Franky Djutanta, Brian Horne, Dominic Showkeir, Robert Rezvani, Chloe Leff, Swechchha Pradhan, and Rizal F. Hariadi
